# Data Augmentation of X-ray Images for Automatic Cargo Inspection of Nuclear Items

**DOI:** 10.3390/s23177537

**Published:** 2023-08-30

**Authors:** Haneol Jang, Chansuh Lee, Hansol Ko, KyungTae Lim

**Affiliations:** 1 Department of Computer Engineering, Hanbat National University, Daejeon 34158, Republic of Korea; hejang@hanbat.ac.kr; 2 Nuclear Export Control Division, Korea Institute of Nuclear Nonproliferation and Control (KINAC), Daejeon 34101, Republic of Korea; chans@kinac.re.kr (C.L.); hsko@kinac.re.kr (H.K.); 3 Department of Nuclear and Quantum Engineering, Korea Advanced Institute of Science and Technology (KAIST), Daejeon 34141, Republic of Korea; 4 Department of Applied Artificial Intelligence, Seoul National University of Science and Technology, Seoul 01811, Republic of Korea

**Keywords:** data augmentation, cargo inspection, semantic segmentation, nuclear items, deep neural networks

## Abstract

As part of establishing a management system to prevent the illegal transfer of nuclear items, automatic nuclear item detection technology is required during customs clearance. However, it is challenging to acquire X-ray images of major nuclear items (e.g., nuclear fuel and gas centrifuges) loaded in cargo with which to train a cargo inspection model. In this work, we propose a new means of data augmentation to alleviate the lack of X-ray training data. The proposed augmentation method generates synthetic X-ray images for the training of semantic segmentation models combining the X-ray images of nuclear items and X-ray cargo background images. To evaluate the effectiveness of the proposed data augmentation technique, we trained representative semantic segmentation models and performed extensive experiments to assess its quantitative and qualitative performance capabilities. Our findings show that multiple item insertions to respond to actual X-ray cargo inspection situations and the resulting occlusion expressions significantly affect the performance of the segmentation models. We believe that this augmentation research will enhance automatic cargo inspections to prevent the illegal transfer of nuclear items at airports and ports.

## 1. Introduction

Security inspections using X-ray images involve the checking of items detected by X-rays by comparing them with a set standard to ensure national security. It is also important for security inspections using X-rays to detect potential risks early in various fields, such as public transportation and events. In the nuclear industry, the need for X-ray-based security inspections is emerging to ensure the accurate detection of major items that can be risk factors [1,2,3,4]. Most X-ray-based identification processes cannot accurately and efficiently identify the target items because many types of objects are often mixed with prohibited items. An inspector should monitor the X-ray images obtained from security inspection devices to detect prohibited items such as guns, ammunition, explosives, toxic substances, and radioactive materials. However, to perform this job, the inspector must focus on the X-ray screen for extended periods, increasing worker fatigue [5,6]. Thus, there is a great demand for a technology that can automatically find objects with special uses and prevent the spread of objects that are prohibited [7,8]. Applying recent deep-learning-based object detection could be one way to solve this problem. However, there is a problem in that recent deep-learning-based technologies for recognizing objects require considerable amounts of high-quality training data.

In order to set up a management system that avoids the unauthorized transfer of nuclear materials, we need technology that can automatically identify key nuclear items during customs checks. However, obtaining X-ray images of such significant nuclear items in cargo for the training of an inspection model presents difficulties. Hence, this research concurs that automatic detection technology specifically for inspecting cargo is necessary and proposes using X-ray image synthesis technology to make up for the shortage of training data.

In this work, we propose a new type of data augmentation to alleviate the lack of X-ray training data. Thus, the goal of this research is to generate training data using data augmentation techniques to combine X-ray images of special-purpose objects with X-ray images of the background. The generated dataset can be used for training a deep-learning-based segmentation model to inspect prohibited items. The main contributions proposed in this study can be summarized as follows. (1) We suggest a new way to generate X-ray data of nuclear items for semantic segmentation. (2) We propose a special-purpose detection system that uses the proposed data augmentation and semantic segmentation algorithm. (3) We conduct extensive experiments using the nuclear item detection system to verify the performance and practicality of the proposed method.

## 2. Related Work

### 2.1. Automatic Cargo Inspection

Due to the recent development of deep-learning-based computer vision technology, AI-based X-ray inspection system development is attracting attention. Object detection and semantic segmentation are applicable computer vision techniques for X-ray inspections. Specifically, semantic segmentation has the advantage of obtaining detailed experimental results compared to object detection, given its ability to undertake pixel-level classification of images.

Representative network structures for semantic segmentation include UNet [9], FPN [10], DeepLabV3+ [11], and HRNet-OCR [12]. UNet is a network structure composed of an encoder that generates representation features and a decoder that generates segmentation results using the representation features. It is characterized by how it combines the feature maps of the decoder and the encoder, which have identical resolutions, to produce good segmentation results. FPN performs well for multi-scale images by performing multi-scale feature fusion using a feature pyramid network structure. DeepLabV3+ uses atrous separable convolution, which combines depth-wise separable convolution and atrous convolution, showing a good segmentation performance while dramatically reducing the number of parameters and the amount of computation. HRNet-OCR is characterized by its ability to generate segmentation results considering object regions by utilizing object-contextual representation, showing state-of-the-art performance on many segmentation benchmarks. However, for this AI-based system, there is a limitation in that high-quality X-ray image datasets of large size must be available to achieve good results [5,13]. In addition, because most of the publicly available X-ray inspection benchmarks are X-ray images for baggage inspection purposes, another problem is that they cannot be used for cargo inspections with different X-ray imaging energy levels [5,14,15,16,17,18,19].

The first study to introduce a publicly available benchmark for cargo inspections was MFA-net [13], which provides the CargoX dataset, compiled by synthesizing prohibited items (e.g., knives) after randomly cropping cargo X-ray images. More specifically, the CargoX dataset is a benchmark for cargo inspections created by inserting prohibited items into random locations. However, because the CargoX dataset does not include X-ray images of nuclear items and only one banned item is synthesized per image, it cannot readily detect overlapping items. Therefore, the CargoX dataset is not suitable for nuclear item detection. In this study, multiple X-ray images of nuclear items are inserted into a cargo X-ray image but randomly rotated, randomly scaled, and randomly located to respond to more diverse scanning environments and item changes.

### 2.2. Data Augmentation for Semantic Segmentation

X-ray datasets of high quality and large size are needed to train a semantic segmentation model for the purpose of X-ray cargo inspections. However, X-ray images from cargo inspections are rare. In particular, X-ray images of nuclear items taken with a cargo inspection scanner and accurate label information are not publicly available. Therefore, in order to prevent the illegal transfer of nuclear items in the future, it is crucial to create a synthetic dataset using data augmentation and train a semantic segmentation model.

Various augmentations for image classification have been introduced [20,21,22,23]. Cutout [22] is a simple regularization method that randomly masks patches from the input image. Cutout allows the model to see the entire area of the input image rather than a specific area. AugMix [23] is a method that improves the robustness and uncertainty and mixes the results of data augmentation in a convex combination to prevent image degradation while maintaining diversity. These augmentations are mainly used for encoding invariances to data transformations and are well-suited for image classification.

Various augmentation methods have been introduced for semantic segmentation, most notably CutMix [24] and Copy-Paste [25]. CutMix [24] replaces the area to be mixed with a patch of another image to solve the problem of Cutout augmentation, significantly reducing the informative pixels of an image. With CutMix, every pixel within the image becomes informative, and the benefits from local dropout are obtained. Copy-Paste [25] is a way to paste objects from one image to another to create many new combinations of training data. These augmentations are used for semantic segmentation of general images, meaning that there is a limitation in that they cannot respond to the X-ray transmittance according to the physical properties of overlapping objects.

To train a segmentation model that performs automatic cargo inspections without cargo X-ray images, a means of synthesizing the textures of backscatter X-ray (BSX) images using a GAN was also introduced [14]. However, in our study, it is assumed that general cargo X-ray images and X-ray images of nuclear items taken by the same high-energy electromagnetic radiation used for cargo inspections are held. Hence, artificial texture generation is not required.

## 3. Method

### 3.1. Proposed X-ray Data Augmentation

**Figure 1:**
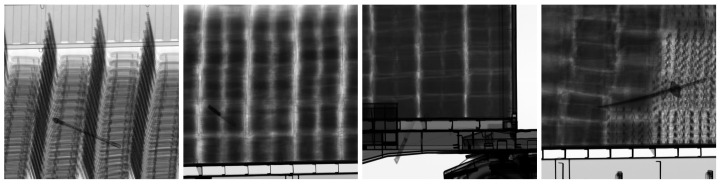
Examples from the CargoX dataset [13].

**Figure 2:**
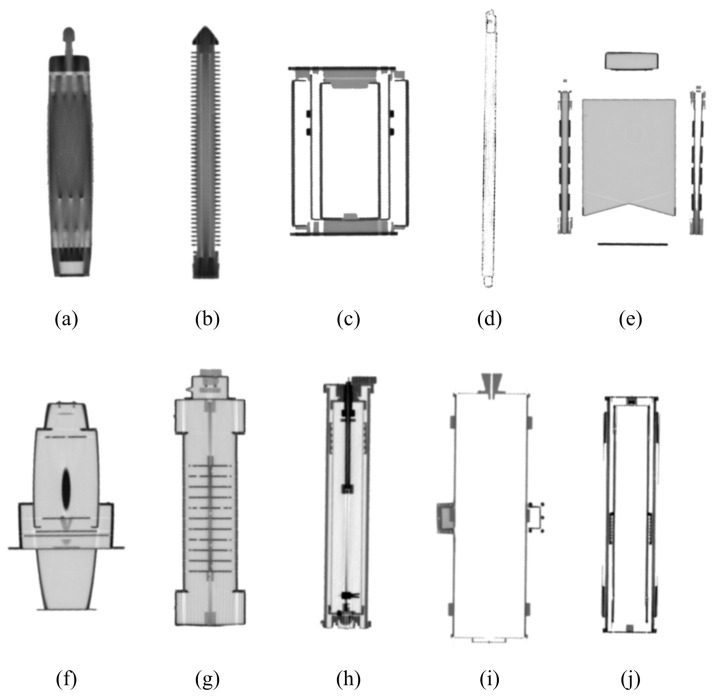
X-ray samples of nuclear items: (**a**) IRT2000 nuclear fuel, (**b**) Magnox nuclear fuel, (**c**) reduction furnace, (**d**) PLUS7 nuclear fuel, (**e**) dissolver, (**f**) mixer–settler, (**g**) pulsed column, (**h**) P4-type gas centrifuge, (**i**) rotary kiln, and (**j**) P2-type gas centrifuge.

The goal is to synthesize X-ray images using data augmentation techniques in this study. The synthesized X-ray images can be utilized to train the segmentation model of major nuclear items in large cargo. We generated X-ray images of major nuclear items in large cargo using both X-ray images of major nuclear items taken at the electromagnetic energy radiation levels used for cargo inspections and randomly cropped X-ray images of large cargo from the CargoX dataset [13]. Figure 1 shows sample X-ray images from the CargoX dataset, where prohibited items are located in large cargo. The CargoX dataset consists of a total of 64,000 X-ray images, and four prohibited items are randomly positioned on the X-ray images. Figure 2 shows examples X-rays of major nuclear items that are actually taken using the same energy of electromagnetic energy radiation used for cargo inspections, courtesy of the Korea Institute of Nuclear Nontransfer And Control (KINAC). In total, ten major nuclear items were used in this study: an IRT2000 nuclear fuel, a Magnox nuclear fuel, a PLUS7 nuclear fuel, a P2-type gas centrifuge, a P4-type gas centrifuge, a pulsed column, a mixer–settler, a reduction furnace, a dissolver, and a rotary kiln.

**Figure 3:**
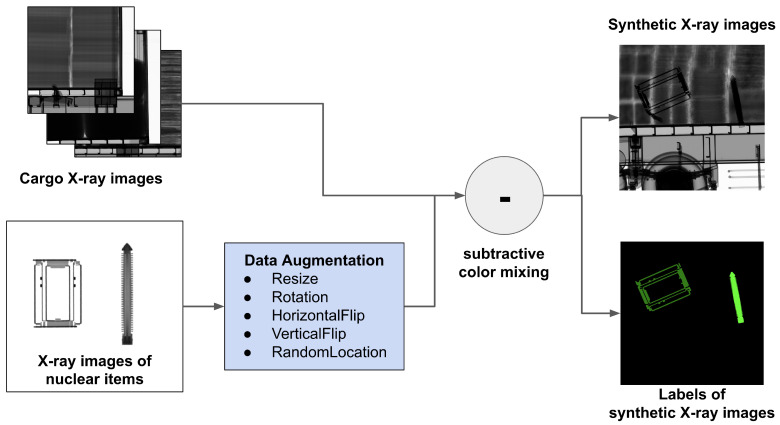
Overall process of the proposed data-generation method.

The process of synthesizing X-ray images to identify major nuclear items is shown in Figure 3. First, we load an X-ray image from CargoX as the background cargo image and an X-ray image of a major nuclear item as the object to be synthesized. We conceived of various nuclear item loading situations and synthesized X-ray images by data-augmenting X-ray images of major nuclear items. When loading the nuclear items, the rotation angle and the location can be changed. Also, when multiple items are loaded in the X-ray image scanning direction, occlusion may occur. In order to meet the requirements of this loading situation, we augmented X-ray images of nuclear items and inserted them into the background cargo X-ray images. The methods we used to augment the X-ray images of nuclear items are resizing, random rotation (from 0 to 90 degrees), horizontal flipping, vertical flipping, and random positioning.

Given that the transmittance of X-ray images decreases as objects overlap, we synthesized X-ray images of nuclear items with subtractive mixing. We inserted up to four nuclear items when generating a synthetic X-ray image, processed subtractive mixing using Equation (1), and clipped the pixel values to have values between 0 and 255.

(1) $${\hat{I}}_{i,j}=\mathrm{clip}\left(I_{i}-I_{j},\;0,\;255\right),$$

where $I_{i}$ , $I_{j}$ , and ${\hat{I}}_{i,j}$ are the *i*-th sample of the CargoX dataset, the *j*-th sample of the nuclear item dataset, and the synthesized X-ray image that is clipped between 0 and 255 after subtracting the *j*-th nuclear item sample from the *i*-th CargoX sample, respectively.

**Figure 4:**
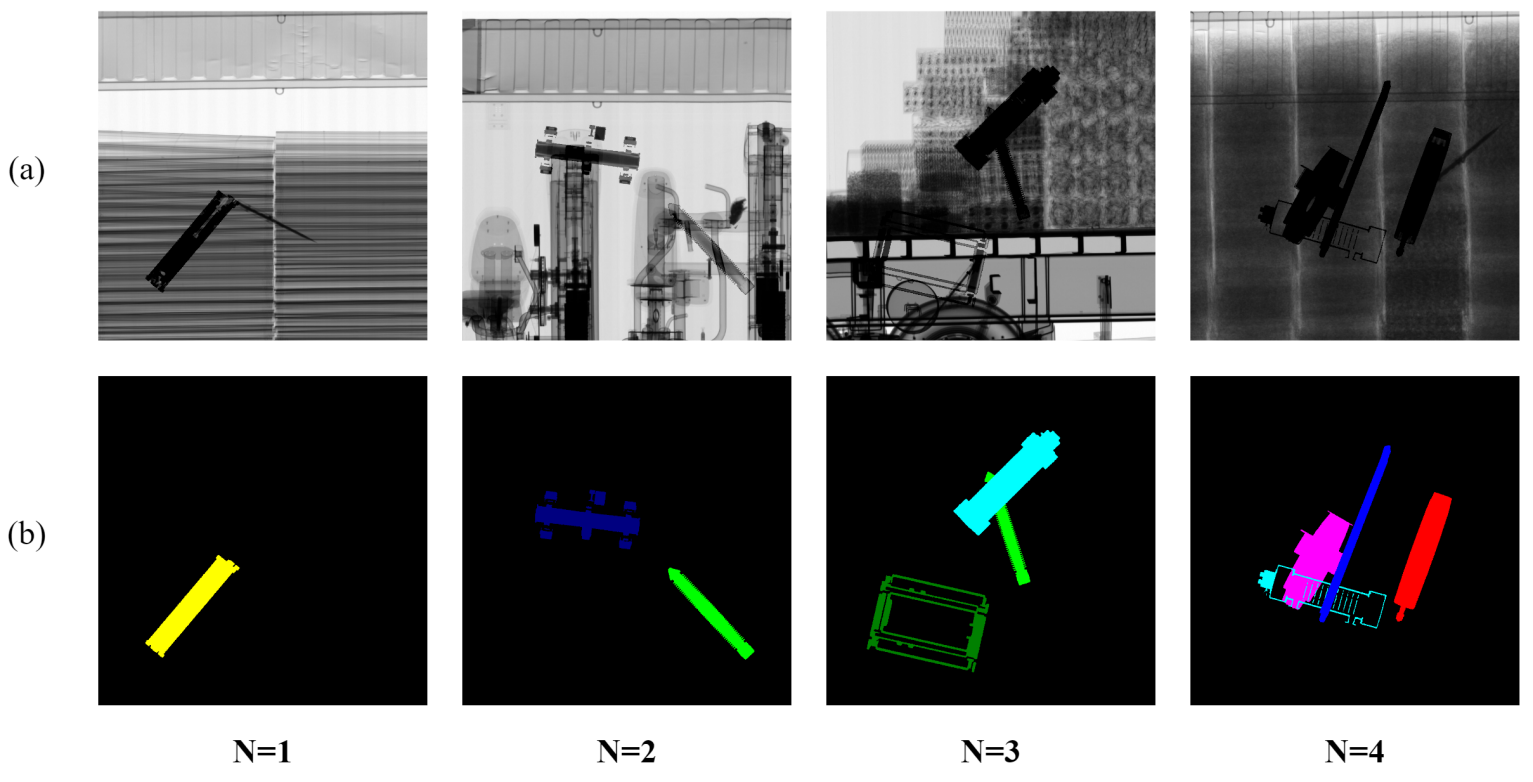
NuclearCargoX examples with changes in the number of nuclear items (N): (**a**) synthetic X-ray images generated using the proposed method and (**b**) corresponding labels for the synthetic X-ray images.

Figure 4 shows examples of NuclearCargoX images built using the proposed augmentation method. The resolution of the NuclearCargoX images is 1024 × 1024 and they are synthesized using X-ray images of ten major nuclear items and the CargoX X-ray images.

### 3.2. Semantic Segmentation for Nuclear Items

**Figure 5:**
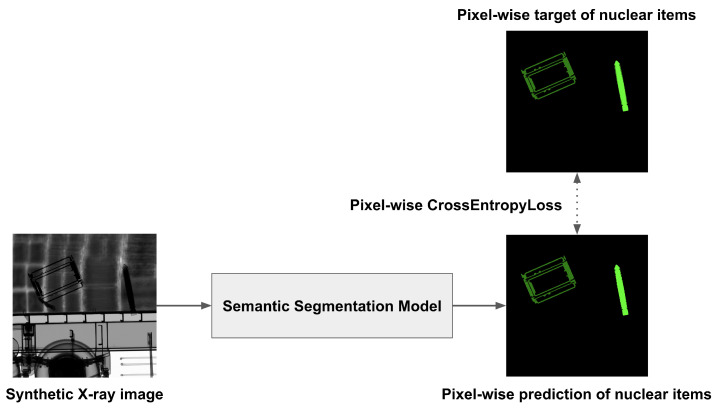
Training process of semantic segmentation on nuclear items.

In this study, we employed a semantic segmentation model to obtain nuclear item detection results in pixel value units. As shown in Figure 5, we set the image and the label of the NuclearCargoX dataset as the input and target values of the semantic segmentation model, respectively. Using Equation (2), the loss in pixel value units is calculated, after which the learnable parameters of the segmentation model are updated.

(2) $$L=\frac{1}{M\cdot P}\sum_{i=1}^{M}\sum_{j=1}^{P}\sum_{k=1}^{K}y_{k}^{\left(i,j\right)}\log\left(\hat{p_{k}^{\left(i,j\right)}}\right).$$

In this equation, *L*, *M*, *P*, and *K* are the cross-entropy loss value in units of pixels, the number of images, the total pixels per image, and the number of item types, respectively. *L* is calculated as the average value of the cross-entropy loss between the target value *y* and the predicted value *p* in pixel value units.

In this study, we used representative semantic segmentation architectures such as UNet [9] and FPN [10], DeepLabV3+ [11], and HRNet-OCR [12]. The effect of the proposed augmentation approach was evaluated using the segmentation models.

## 4. Experiments

### 4.1. NuclearCargoX Dataset

The NuclearCargoX dataset contains 20,000 X-ray images of nuclear items and labels corresponding to the X-ray images synthesized using the proposed augmentation methods. The image resolution is 1024 × 1024. A total of ten nuclear items was used in the NuclearCargoX dataset, and two X-ray images that most feasibly characterized each item were taken. The list of nuclear items is as follows: an IRT2000 nuclear fuel, a Magnox nuclear fuel, a PLUS7 nuclear fuel, a P2-type gas centrifuge, a P4-type gas centrifuge, a pulsed column, a mixer–settler, a reduction furnace, a dissolver, and a rotary kiln. The NuclearCargoX dataset was divided as follows: 10,000 images for training, 5000 images for validation, and 5000 images for testing. We generated labelled images by expressing the ten major nuclear items in different colors. In order to replicate various loading situations, we set the maximum number of inserted nuclear items to 1, 2, 3, and 4, after which a dataset of the maximum number of each nuclear item was generated. A random position was cropped from the original size of 1024 × 1024 to a size of 512 × 512 for training and the full size of 1024 × 1024 was used for validation and testing. The data augmentation method proposed in this study can simultaneously synthesize various nuclear items and indicate when the transmittance is lowered due to item occlusion. We synthesized the images using subtractive mixing of the pixel values of the overlapping nuclear items, similar to overlapping items in actual X-ray images.

### 4.2. Evaluation Metrics

The mean intersection over union (mIoU) and the pixel accuracy are used as evaluation indicators for detecting major nuclear items. mIoU is the average of IoUs of all classes, and IoU is the area of overlap between the predicted segmentation and the ground truth divided by the area of union between the predicted segmentation and the ground truth. Pixel accuracy refers to the percentage of pixels in an image classified correctly. The mIoU and pixel accuracy equations are presented here as Equations (3) and (4), respectively.

(3) $$\mathrm{mIoU}=\frac{1}{K}\sum_{k=1}^{K}\frac{TP_{k}}{TP_{k}+FP_{k}+FN_{k}},$$

(4) $$\mathrm{pixel}\;\mathrm{accuracy}=\frac{TP}{TP+TN+FP+FN}.$$

In this equation, *K* is the number of nuclear item classes. True positive (*TP*) is the number of pixels where both the target and predicted values are positive, and true negative (*TN*) is the number of pixels where both the target and predicted values are negative. False positive (*FP*) is the number of pixels with a negative target and positive prediction, and false negative (*FN*) is the number of pixels with a positive target and negative prediction.

### 4.3. Implementation Details

Given that there are relatively few X-ray images of nuclear items, there is a problem in that the diversity of X-ray images is limited, even when we synthesize the X-ray images. To overcome this problem, we fine-tuned the segmentation model pretrained with the ImageNet benchmark. The number of nuclear item classes *K* was set to 11 for segmentation of the background and ten nuclear items. For stable model training, the polynomial learning rate scheduler was used, and the learning rate dropped with the polynomial from the initial learning rate of $10^{-3}$ to the final learning rate of $10^{-6}$ . The weight decay was set to $5\;\times \;10^{-4}$ , and AdamW [26] was used as the optimization function. The mini-batch size was set to 16. The maximum number of training iterations was set to 20 because the performance convergence of the segmentation models occurred before 20 training iterations. All hyper-parameters for training were set to the optimal values obtained experimentally.

## 5. Results

### 5.1. Quantitative Performance

#### Performance of the Proposed Data Augmentation Method

To investigate the generalization performance in relation to the change in the maximum number (N) of nuclear items synthesized in an X-ray image, we conducted an experiment in which $N_{train}$ and $N_{test}$ were both changed from 1 to 4. Table 1 and Table 2 indicate the mIoU and the pixel accuracy outcomes, respectively, of the various augmentations. Among the augmentation methods, the proposed method showed the best mIoU and pixel accuracy for all segmentation models. All augmentation methods performed best on the UNet segmentation model. Table 3 and Table 4 indicate the mIoU and the pixel accuracy outcomes, respectively, of the proposed augmentations with the four segmentation models. For the UNet models, as $N_{train}$ was increased, the average performance improved and the gap in the performance improvement gradually decreased. UNet with $N_{train}=3$ showed the best performance, and the model with $N_{train}=4$ showed a slightly lower performance. The HRNet-OCR model, the heaviest among the semantic segmentation models used in the experiment, did not perform as well as the UNet model, specifically showing the largest mIoU difference (11.16%) compared to UNet with $N_{train}=1$ .

**Figure 6:**
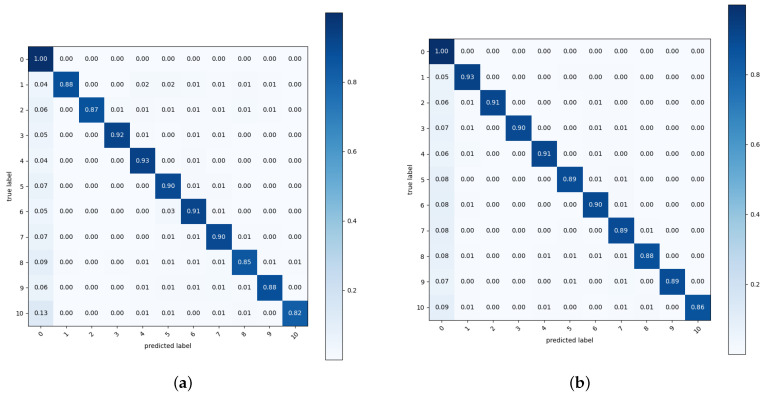
Confusion matrix of the test pixel accuracy where $N_{train}=4$ and $N_{test}=4$ : (**a**) Copy-Paste [25] and (**b**) ours. The labels 0 to 10 respectively denote the background, IRT2000 nuclear fuel, Magnox nuclear fuel, PLUS7 nuclear fuel, P4-type gas centrifuge, pulsed column, mixer–settler, dissolver, reduction furnace, rotary kiln, and P2-type gas centrifuge.

Figure 6 shows the confusion matrices of the test pixel accuracy of Copy-Paste and the proposed augmentation. We evaluated the test pixel accuracy for classes of the background (0) and nuclear items (1 to 10). In all augmentations, the rate of incorrectly predicting nuclear items as the background was higher than the prediction errors between nuclear items. Among nuclear items, the P2-type gas centrifuge, where the class number was 10, showed many errors in both Copy-Paste and the proposed augmentation. As depicted in Figure 2, the P2-type gas centrifuge has a high transmittance in most areas, so the amount of informative pixels used for training segmentation models is small. This lack of informative pixels seems to cause a high error rate.

### 5.2. Qualitative Performance

**Figure 7:**
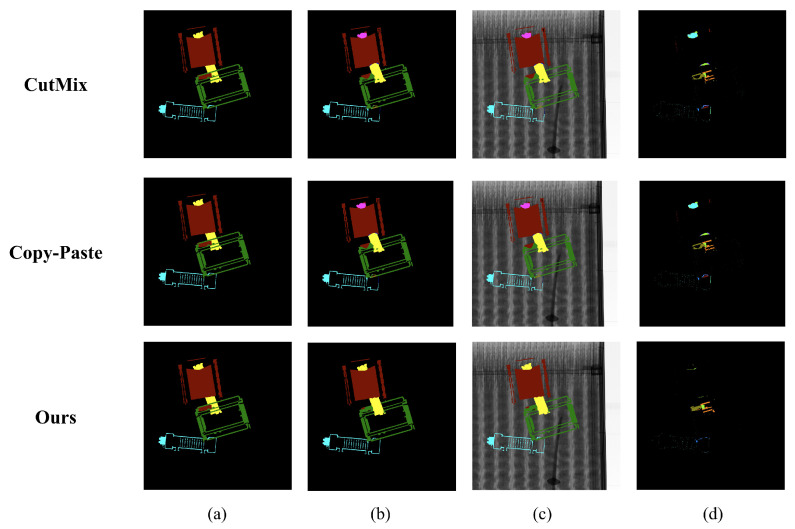
Visualization of the segmentation results according to augmentation changes where $N_{train}=4$ and $N_{test}=4$ : (**a**) ground truths, (**b**) prediction results, (**c**) prediction overlay images, and (**d**) differences between ground truths and predictions.

**Figure A1:**
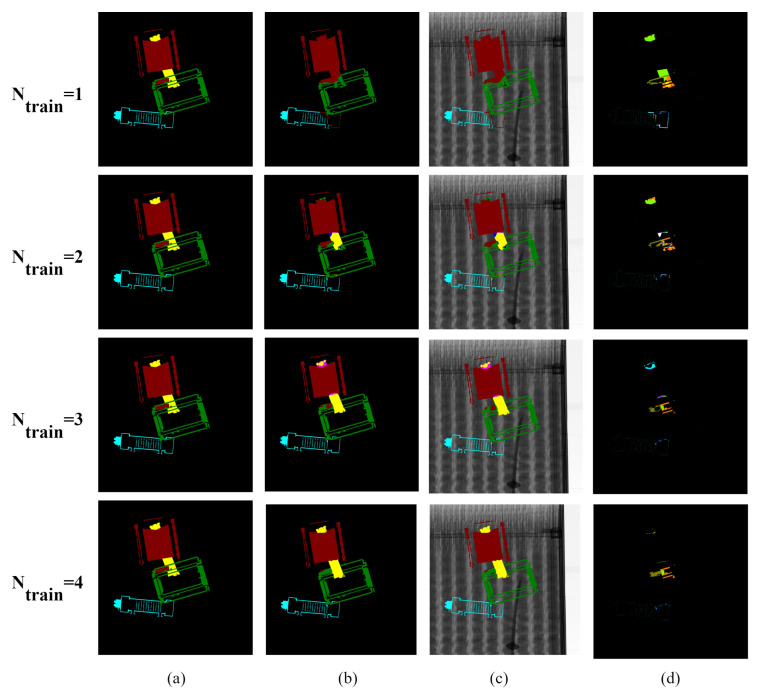
Visualization of the UNet segmentation results according to $N_{train}$ changes: (**a**) ground truths, (**b**) prediction results, (**c**) prediction overlay images, and (**d**) differences between ground truths and predictions.

**Figure A2:**
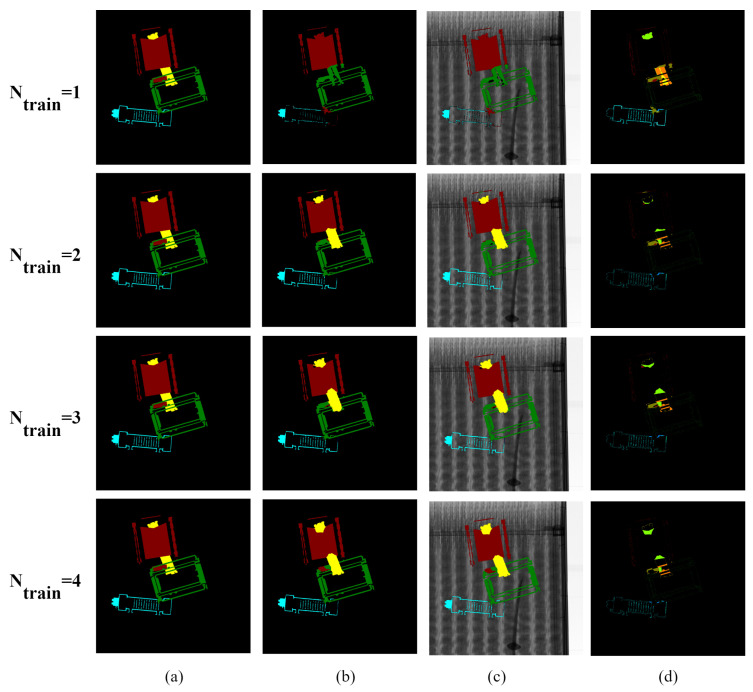
Visualization of the FPN segmentation results according to $N_{train}$ changes: (**a**) ground truths, (**b**) prediction results, (**c**) prediction overlay images, and (**d**) differences between ground truths and predictions.

**Figure A3:**
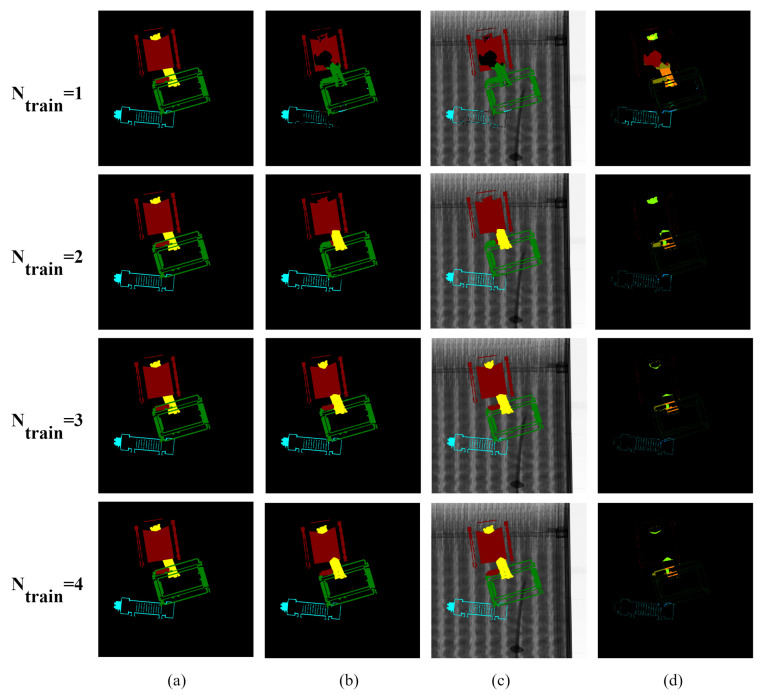
Visualization of the DeepLabV3+ segmentation results according to $N_{train}$ changes: (**a**) ground truths, (**b**) prediction results, (**c**) prediction overlay images, and (**d**) differences between ground truths and predictions.

**Figure A4:**
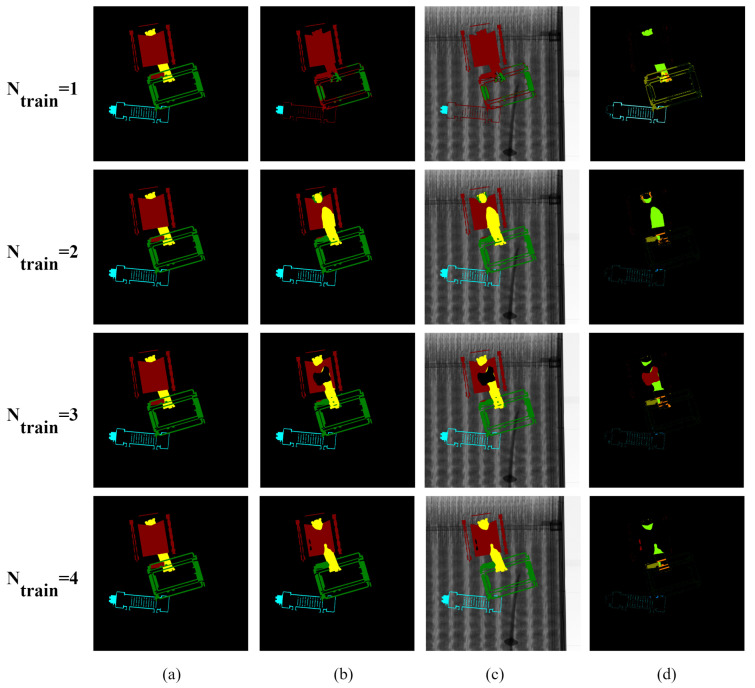
Visualization of the HRNet-OCR segmentation results according to $N_{train}$ changes: (**a**) ground truths, (**b**) prediction results, (**c**) prediction overlay images, and (**d**) differences between ground truths and predictions.

We visualized the UNet segmentation results to compare the qualitative performance of the augmentations. Figure 7 illustrates the visualization comparison results of the augmentations used in the experiment. As observed in Table 1 and Table 2, which show the quantitative performance outcomes, the UNet model with the proposed augmentation showed the least error in the visualization results. Figure A1, Figure A2, Figure A3 and Figure A4 visualize the segmentation results of the proposed augmentation with the UNet, FPN, DeepLabV3+, and HRNet-OCR, respectively.

**Figure 8:**
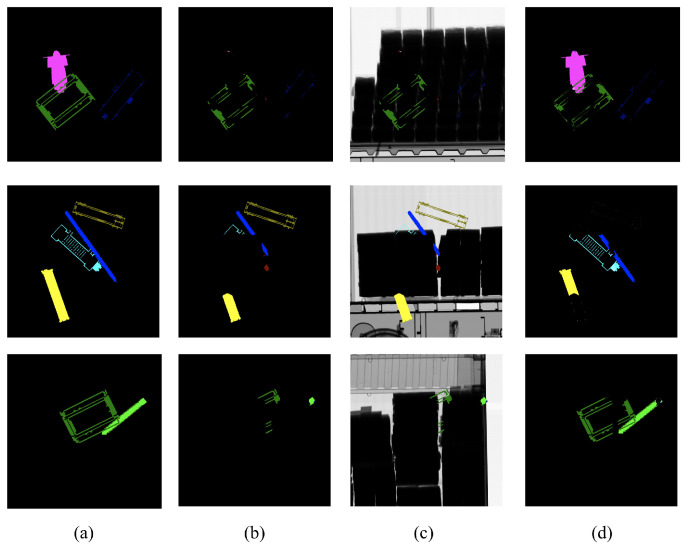
Visualization of the failure cases of the proposed augmentation where $N_{train}=4$ and $N_{test}=4$ : (**a**) ground truths, (**b**) prediction results, (**c**) prediction overlay images, and (**d**) differences between ground truths and predictions.

Figure 8 shows the visualization of the failure cases of the proposed augmentation. Since X-ray images represent the transmittance of the physical properties of the captured object, the denser the object and the greater the number of overlaps, the lower the transmittance and the closer it is to zero. In all cases of failure, the transmittance was extremely low and the pixel values of the X-ray image were close to zero.

## 6. Discussion and Conclusions

It is crucial at present to develop a semantic segmentation technique for X-ray cargo inspections in order to prevent the illegal transfer of nuclear items. While X-ray datasets of good quality and a large size are required to train a deep-learning-based segmentation model, X-ray images from cargo inspections are rare. Specifically, X-ray images of nuclear items loaded into a cargo are almost impossible to obtain.

In this study, we proposed an augmentation method to mitigate the problem of the lack of data in the X-ray cargo inspection field. Unlike representative augmentations such as Cutout, CutMix, AugMix, and Copy-Paste, widely used in computer vision, the proposed augmentation method can express the X-ray transmittance according to the physical properties and the transmittance of overlapping objects. This data augmentation technique is highly scalable in that it can be applied not only to the nuclear field but also to special-purpose identification fields where acquiring training data is very difficult. To respond to various X-ray cargo inspection situations, we augmented the X-ray images of nuclear items in various ways (i.e., resizing, flipping, random rotation, random location, and multiple insertions) to generate the NuclearCargoX dataset. We also trained representative segmentation models using NuclearCargoX and evaluated the quantitative and qualitative performance outcomes. The experiment results reveal that the proposed augmentation is feasible for use in cargo X-ray inspection. However, the proposed augmentation does not perform well in cases of extremely low transmittance (high density of objects or high overlap of objects), so manual inspection of the cargo may be required in such cases. Furthermore, when an object has multiple separate components, such as object (e) in Figure 2, each component may be interpreted as a different object, leading to inaccurate semantic segmentation. This research addresses data augmentation methods to improve the performance of semantic segmentation of nuclear items in the limited situation where only X-ray images of nuclear items and cargo are accessible, i.e., the normalized intensity of each object is not accessible. In further research, if we have access to the normalized intensity of each object in the cargo and nuclear items, we will mathematically represent the X-ray transmission in exponential form and design more sophisticated data augmentation.

As in this study, in a situation where there are relatively few X-ray images of nuclear items, even if the dataset is augmented, the diversity of training data that can be generated is limited. We believe this augmentation research will enhance automatic cargo inspections, furthering efforts to prevent the illegal transfer of nuclear items at airports and ports.

## Figures and Tables

**Table 1:** mIoU (%) of various augmentations with the four segmentation models where $N_{train}=4$ and $N_{test}=all$ .

Augmentation	UNet	FPN	DeepLabV3+	HRNet−OCR
CutMix [24] (2019)	89.19	84.98	85.52	79.35
Copy-Paste [25] (2021)	89.19	86.65	83.64	84.77
Ours	90.77	88.45	88.41	87.28

**Table 2:** Pixel accuracy (%) of various augmentations with the four segmentation models where $N_{train}=4$ and $N_{test}=all$ .

Augmentation	UNet	FPN	DeepLabV3+	HRNet−OCR
CutMix [24] (2019)	99.67	99.52	99.53	99.31
Copy-Paste [25] (2021)	99.67	99.56	99.46	99.46
Ours	99.69	99.62	99.62	99.55

**Table 3:** mIoU (%) of the proposed augmentation according to the change in the number of nuclear items in an image used for training and testing.

Model	$N_{\mathrm{test}}$	$N_{\mathrm{train}}=1$	$N_{\mathrm{train}}=2$	$N_{\mathrm{train}}=3$	$N_{\mathrm{train}}=4$
UNet	1	92.41	93.47	94.53	93.54
	2	83.75	90.95	92.45	91.72
	3	76.51	88.83	90.51	89.93
	4	69.88	86.04	88.23	87.89
	Average	80.63	89.82	91.43	90.77
FPN	1	88.49	89.52	89.05	90.98
	2	79.07	87.57	87.35	89.24
	3	72.70	85.66	85.72	87.79
	4	66.06	83.35	83.59	85.78
	Average	76.58	86.53	86.43	88.45
DeepLabV3+	1	90.84	89.77	91.08	90.99
	2	82.46	87.64	89.24	89.26
	3	73.91	85.63	87.57	87.71
	4	64.51	83.05	85.32	85.66
	Average	77.93	86.52	88.30	88.41
HRNet-OCR	1	91.60	90.62	90.61	89.50
	2	70.71	90.14	88.90	88.16
	3	60.93	89.20	87.19	86.61
	4	54.63	86.97	85.23	84.83
	Average	69.47	89.23	87.98	87.28

**Table 4:** Pixel accuracy (%) of the proposed augmentation according to the change in the number of nuclear items in an image used for training and testing.

Model	$N_{\mathrm{test}}$	$N_{\mathrm{train}}=1$	$N_{\mathrm{train}}=2$	$N_{\mathrm{train}}=3$	$N_{\mathrm{train}}=4$
UNet	1	99.84	99.87	99.88	99.86
	2	99.61	99.76	99.79	99.77
	3	99.27	99.64	99.68	99.65
	4	98.83	99.45	99.53	99.49
	Average	99.39	99.68	99.72	99.69
FPN	1	99.76	99.78	99.95	99.82
	2	99.45	99.65	99.64	99.70
	3	99.08	99.49	99.49	99.58
	4	98.56	99.28	99.28	99.40
	Average	99.21	99.55	99.59	99.63
DeepLabV3+	1	99.82	99.79	99.82	99.82
	2	99.56	99.65	99.71	99.70
	3	99.16	99.49	99.57	99.57
	4	98.52	99.28	99.39	99.40
	Average	99.26	99.55	99.62	99.62
HRNet-OCR	1	99.83	99.80	99.80	99.77
	2	99.32	99.72	99.67	99.64
	3	98.80	99.64	99.52	99.49
	4	98.18	99.48	99.33	99.30
	Average	99.03	99.66	99.58	99.55

## Data Availability

The data used in this study can be made available upon reasonable request.

## Abbreviations

The following abbreviations are used in this manuscript:

NEPS	Nuclear export and import control system
BSX	Backscatter X-ray
GAN	Generative adversarial network
Params	The number of learnable parameters
FLOPs	Floating-point operations

## Appendix A. Visualization of the Segmentation Results

Figure A1, Figure A2, Figure A3 and Figure A4 visualize the segmentation results of the proposed augmentation with the UNet, FPN, DeepLabV3+, and HRNet-OCR, respectively.
